# Anatomical Characteristics of the Accessory Maxillary Ostium in Three-Dimensional Analysis

**DOI:** 10.3390/medicina58091243

**Published:** 2022-09-08

**Authors:** Jiwon Do, Jeong Joon Han

**Affiliations:** 1 Department of Oral and Maxillofacial Surgery, School of Dentistry, Dental Research Institute, Seoul National University, Seoul 03080, Korea; jiwon9406082@gmail.com; 2 Department of Oral and Maxillofacial Surgery, Seoul National University Dental Hospital, Seoul 03080, Korea

**Keywords:** accessory maxillary ostium, natural ostium, three-dimensional analysis, anatomy, sinus pathology

## Abstract

*Background and Objectives*: The accessory maxillary ostium (AMO) can interfere with ventilation and drainage of the maxillary sinus, and therefore the importance of evaluating the anatomical features of the AMO has been emphasized. This study aimed to evaluate anatomical characteristics of the AMO together with the natural ostium (NO) using three-dimensional (3D) analysis and to assess the relationship between the AMO and maxillary sinus pathologies. *Materials and Methods*: This retrospective study included 394 sinuses in 197 patients. Using 3D computed tomography images, the prevalence of the AMO and concurrent sinus pathologies were examined. For patients with an AMO, 3D spatial positions of the AMO and NO related to adjacent anatomic structures and dimensions of the AMO and NO were evaluated. *Results*: A total of 84 sinuses showed single or multiple AMO, with a prevalence of 21.3%. The AMO was located superiorly by 30.1 mm from the maxillary sinus floor, inferiorly by 1.3 mm from the orbital floor, and posteriorly by 22.4 mm from the anterior sinus wall. The AMO was located 5.4 mm posteriorly and 0.7 mm inferiorly from the NO. On the same coronal plane as the NO or AMO, height from the maxillary sinus floor to the NO and AMO ranged from 19.4 to 45.8 mm and 14.5 mm to 41.9 mm, respectively. The mean horizontal and vertical dimensions were 5.9 mm and 4.6 mm for the NO and 2.8 mm and 3.0 mm for the AMO. We detected a significant association between the presence of the AMO and the mucosal thickening (*p* = 0.029). *Conclusions*: The results of this study suggest that, although the AMO and NO are mostly located in positions that do not limit sinus-related surgeries, such as maxillary sinus floor augmentation, the AMO and NO are also found in lower positions, which may be a detriment to the postoperative physiological function of the maxillary sinus and affect treatment outcomes.

## 1. Introduction

The maxillary sinus is a pyramid-shaped bony cavity lined with sinus membrane, also known as the Schneiderian membrane. This membrane is continuous with the nasal epithelium through the natural ostium (NO) in the middle meatus. The NO is responsible for drainage and ventilation of the sinus and maintains the physiological condition of the maxillary sinus [1]. The NO is located on the superomedial aspect of the maxillary sinus, and the blockage of the NO may result in pathologic conditions of the maxillary sinus [2].

The accessory maxillary ostium (AMO) is an anatomical variation located between the uncinate process and the inferior concha [3,4]. Although several investigators have showed results supporting that the AMO are maxillary sinus perforations in the fontanelles caused from maxillary sinusitis, it is still unknown whether the AMO is a congenital anatomical variation or an acquired defect [5,6]. When present, the AMO is thought to disturb the physiologic functions of the maxillary sinus and contribute to pathologic conditions of the sinus [3,7,8]. The existence of the AMO can be associated with mucous recirculation, or re-drainage from the nasal cavity to the sinus or the NO to the AMO, although not in all sinuses with an AMO [9]. In the study by Mladina, et al. [6], the circulating mucus ring between two ostia was observed in 9.2% of patients with an AMO. Several previous studies suggested that mucous recirculation from the AMO causes chronic persistent maxillary sinusitis [5,10,11]. The AMO can also markedly increase the sinus ventilation and change the pattern of airflow, thereby lowering nitric oxide concentration in the sinus [12]. Considering that nitric oxide provides an antibacterial environment for the paranasal sinuses and regulates the mucociliary clearance function of the sinus membrane, a decrease in nitric oxide concentration may lead to maxillary sinusitis [12,13,14].

With the development of endoscopic technology, endoscopic sinus surgery (ESS) has been widely used for the treatment of maxillary sinus pathologies, including maxillary sinusitis. In ESS, the NO is usually widened to promote ventilation and drainage from the sinus to the nasal cavity, with removal of the anatomic variants that can interfere with the patency of the NO. Since there are important adjacent anatomical structures around the NO, such as the orbital floor and lacrimal gland, a lack of understanding of the anatomic features of the maxillary sinus and its adjacent structures could lead to fatal complications upon injury [15,16]. Furthermore, when the surgeon cannot locate the NO correctly due to incomplete removal of the inferior part of the uncinate process and performs middle meatal antrostomy posterior to the NO, a subsequent missed ostium sequence may occur [10,11]. Mucous recirculation between the NO and the surgically created AMO could then occur, leading to the failure of ESS.

In other sinus-related surgeries in the oral and maxillofacial region, such as dental implant surgery with maxillary sinus floor augmentation (MSFA), apical surgery for endo-periosteal lesion reaching the maxillary sinus, extraction of impacted teeth, and odontogenic maxillary sinus surgery, it is essential to understand the anatomic features of the maxillary sinus to accurately establish treatment plans, safely and successfully perform surgical procedures, and prevent potential postoperative complications [1].

Previously, the locations and diameters of the NO and AMO were established through cadaveric study, endoscopic examination, or computed tomography (CT)/cone-beam computed tomography (CBCT) analysis [3,4,8,17]. However, in cadaveric study, post-mortem distortion of anatomic structures may hinder accurate measurements and analysis, and it is difficult to obtain a large study sample. In endoscopic examinations, although a hiatus semilunaris can be seen, it is not easy to examine the NO and find the posterior fontanelle perforation. In addition, it is difficult to accurately measure the location and dimension of the NO and AMO. Although anatomic characteristics of the NO and AMO have been investigated using CT analysis, most previous studies examined only single sectional images on each coronal, axial, or sagittal plane and performed measurements related to the location and dimensions of the NO or AMO [18,19,20]. In addition, to our knowledge, few reports have studied the spatial relationships of the NO or AMO with adjacent anatomic landmarks. The aim of this study is to evaluate anatomical characteristics of the AMO and NO using three-dimensional (3D) analysis and to assess the relationship between the presence of the AMO and maxillary sinus pathologies.

## 2. Materials and Methods

### 2.1. Study Design and Sample

We designed and implemented a retrospective study. The study population included all consecutive patients who underwent 3D CT scanning from January 2019 to December 2021 at the Department of Oral and Maxillofacial Surgery, Seoul National University Dental Hospital. Given its retrospective nature and de-identifying the records of patients before the start of the study, this study was exempted from approval of the institutional review board of Seoul National University Dental Hospital (ERI22029). The exclusion criteria were as follows: (1) younger than 18 years old; (2) history of previous surgeries or trauma in the area of the maxillary sinus; (3) cleft lip and/or palate; (4) craniofacial syndromes effecting the midface; (5) pathological destruction of the maxillary sinus caused by tumor, osteomyelitis, medication-osteonecrosis of the jaw, or osteoradionecrosis; and (6) all paranasal sinuses not entirely visible on CT images.

### 2.2. Data Acquisition

**Figure 1:**
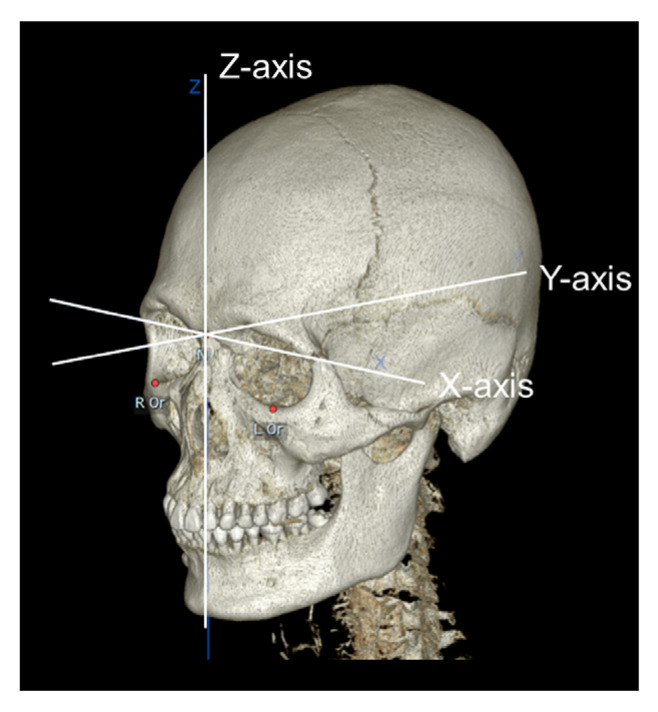
The three-dimensional coordinate system (*X*, *Y*, *Z*) used in this study.

**Figure 2:**
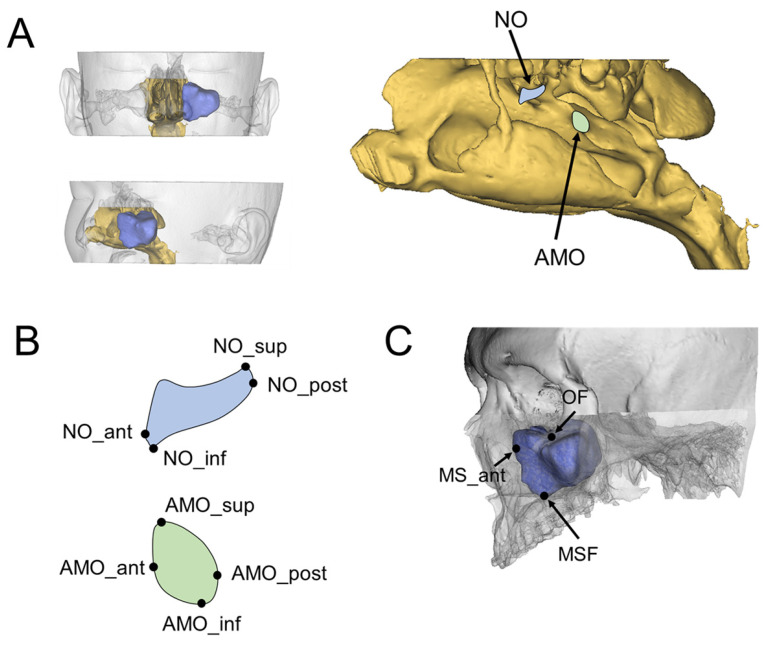
Determination of landmarks for the natural ostium and accessory maxillary ostium. (**A**) Three-dimensionally constructed nasal airway and left maxillary sinus. The natural ostium and accessory maxillary ostium can be found from the lateral view of the nasal airway. (**B**,**C**) Landmarks of the natural ostium, accessory maxillary ostium, and adjacent anatomic structures. After three-dimensional coordinates (*x*, *y*, *z*) for each landmark were obtained, the horizontal and vertical dimensions of the natural ostium and accessory maxillary ostium and the spatial position of the natural ostium and accessory maxillary ostium related to the adjacent anatomic landmarks, such as the maxillary sinus floor, anterior maxillary sinus wall, and orbital floor, were obtained. The definitions of each landmark are described in Table 1. NO, natural ostium; AMO, accessory maxillary ostium.

**Figure 3:**
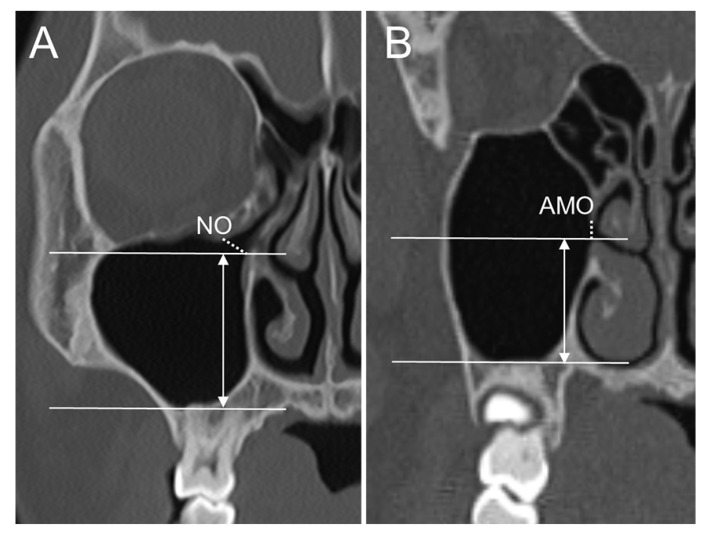
Measurement of the distance between the natural ostium (**A**) or accessory maxillary ostium (**B**) and the maxillary sinus floor located directly below the natural ostium or accessory maxillary ostium. The distance between the most inferior point of the natural ostium or accessory maxillary ostium and the maxillary sinus floor was measured on the same coronal plane as the inferior point of the natural ostium or accessory maxillary ostium.

All 3D CT images (SOMATOM Sensation 10, Siemens, Munich, Germany) were obtained at 120 kVp and 100 mA and saved in digital imaging and communications in medicine (DICOM) format. Using a 3D CT viewer (OnDemand3D viewer, Cybermed, Seoul, Korea), we examined sinuses with an AMO. To determine the relationships between the presence of an AMO and maxillary sinus pathologies, pathologic changes of the maxillary sinus were examined and categorized as mucosal thickening, mucous retention cysts, and maxillary sinusitis. Maxillary sinusitis was defined as a maxillary sinus with an air–fluid meniscus, air bubbles in the fluid, or total opacification of the sinus lumen [14,21]. CT images for patients with a unilateral or bilateral AMO were imported to 3D analysis software (OnDemand3D). The CT analyses were performed by a single investigator trained to accurately assess the anatomic structures and variations of the maxillary sinus. A 3D coordinate system (*X*, *Y*, *Z*) was established for evaluation of the 3D anatomic location of the AMO and NO (Figure 1) [22]. The Frankfort Horizontal plane (FH plane), which passes through the right porion and both orbitale, was defined as the horizontal reference plane. The midsagittal plane was defined as the plane perpendicular to the FH plane and passing through the nasion. The coronal plane was defined as the plane that passed through both orbitale and perpendicular to the FH plane. The anatomic landmarks and measurements are shown in Table 1. Briefly, the most superior, inferior, anterior, and posterior points of the AMO and NO; the most inferior point of the maxillary sinus floor; the most anterior point of the maxillary sinus wall; and the most inferior point of the orbital floor were selected based on the three coronal, axial, and sagittal planes (Figure 2). Using the *x*-, *y*-, and *z*-coordinates obtained for each landmark, the antero-posterior and supero-inferior positions of the AMO and NO related to the adjacent anatomic structures, the spatial relationship between the AMO and NO, and the horizontal and vertical dimensions of the AMO and NO were calculated. The antero-posterior positions of the AMO and NO were also evaluated based on the dentition using the sagittal view of the computed tomography images. To evaluate the distance between the AMO and the maxillary sinus floor located directly below the AMO for each sinus, the height between the most inferior point of the AMO and the maxillary sinus floor was measured on the same coronal plane as the inferior point of the AMO (Figure 3). For the NO, the distance from the maxillary sinus floor located directly below the NO was measured in the same way.

### 2.3. Statistical Analysis

Statistical analysis was performed using SPSS software version 25.0 (IBM Inc., Chicago, IL, USA). The Kolmogorov–Smirnov test was performed to determine whether the data had a normal distribution. Differences in demographic data according to the presence of an AMO were analyzed through the chi-square test or the Mann–Whitney U test. The relationships between maxillary sinus pathologies (mucosal thickening, mucous retention cyst, and maxillary sinusitis) and the presence of an AMO were analyzed using the chi-square test or Fisher’s exact test. The locations and dimensions of the AMO and NO, which were calculated using 3D coordinates (*x*, *y*, *z*), are presented as the mean with standard deviation, and differences depending on sex were analyzed using the paired *t* test. The presence of sinus pathologies according to the presence of an AMO was analyzed using the chi-square test. To determine the factors influencing the presence of sinus pathologies in sinuses with an AMO, a binary logistic regression analysis was conducted for variables including age, sex, horizontal and vertical dimensions of the AMO, and antero-posterior and supero-inferior distance of the AMO from the NO. The statistical significance level was set at *p* < 0.05.

## 3. Results

A total of 197 patients (male:female = 97:100; mean age, 36.7 ± 17.3 years; age range, 18–88 years) who met the inclusion and exclusion criteria were included in the study sample (Table 2). Sixty-three patients (male:female = 28:35; mean age, 37.7 ± 16.7 years, age range, 18–77 years) had an AMO, with a prevalence of 32.0%, where forty-two and twenty-one patients exhibited the AMO unilaterally and bilaterally, respectively. Of 394 sinuses in 197 patients, 84 sinuses exhibited single or multiple AMO, indicating the prevalence of the AMO was 21.3% (Table 3). Six sinuses showed two AMO in each sinus with a prevalence of 7.1% among eighty-four sinuses with an AMO. Five were male and one was female, and all were left-sided AMO. Four sinuses in three patients showed obstruction of the NO due to mucosal thickening (three sinuses) and maxillary sinusitis (one sinus), and these sinuses were excluded in the analysis of the location and dimensions of the NO.

The NO was located superiorly by 33.8 ± 4.6 mm from the maxillary sinus floor, superiorly by 3.2 ± 3.0 mm from the orbital floor, and posteriorly by 11.4 ± 2.7 mm from the anterior maxillary sinus wall (Table 4). The AMO was located superiorly by 30.1 ± 5.0 mm from the maxillary sinus floor, inferiorly by 1.3 ± 3.7 mm from the orbital floor, and posteriorly by 22.4 ± 4.0 mm from the anterior sinus wall. When comparing positions between the NO and AMO, the anterior point of the AMO was located 5.4 ± 4.6 mm posteriorly from the posterior point of the NO, and the superior point of the AMO was located 0.7 ± 3.4 mm inferiorly from the inferior point of the NO. Both the NO and AMO were found to be located significantly superiorly in males than in females vertically, and the NO was found to be located significantly posteriorly in males than in females antero-posteriorly. Other measurements of position did not show significant differences according to sex. In terms of the antero-posterior position of the NO and AMO related to the dentition, 73 NO (59.8%) were located above the second premolar, 36 (29.5%) above the first molar, 12 (9.8%) above the first premolar, and 1 (0.8%) above the second molar. In contrast, more than half of the total AMO were located above the second molar (49 of 90, 54.4%), followed by the first molar (37 of 90, 41.1%) and second premolar (4 of 90, 4.4%). No AMO were anterior to the second premolar. On the same coronal plane as the NO or AMO, the heights from the maxillary sinus floor to the NO and AMO were 27.3 ± 5.5 mm and 31.6 ± 4.9 mm above, respectively, and they ranged from 19.4 to 45.8 mm and 14.5 mm to 41.9 mm, respectively.

With respect to the dimensions of the ostium, the mean horizontal and vertical dimensions of the NO were 5.9 ± 2.8 mm and 4.6 ± 1.9 mm, and the horizontal dimensions were significantly greater than the vertical dimensions (*p* < 0.001). The AMO exhibited horizontal and vertical dimensions of 2.8 ± 1.3 mm and 3.0 ± 1.2 mm, respectively, with no significant difference between the horizontal and vertical dimensions (*p* = 0.170). Although the mean values of antero-posterior and supero-inferior dimensions in the AMO were less than 3.0 mm, they ranged to a maximum of 8.4 mm and 7.1 mm, respectively. There were no significant differences in the horizontal or vertical dimensions of the NO and AMO according to sex (NO, *p* = 0.844 for the horizontal dimension and *p* = 0.676 for the vertical dimension; AMO, *p* = 0.347 for the horizontal dimension and *p* = 0.694 for the vertical dimension).

In terms of concurrent maxillary sinus pathologies, 150 of 394 sinuses (38.1%) exhibited sinus pathologies, which included 104 cases of mucosal thickening, 48 mucous retention cysts, and eight cases of maxillary sinusitis. Ten sinuses showed two types of sinus pathologies within each sinus. Of the 84 sinuses with an AMO, mucosal thickening, a mucous retention cyst, and maxillary sinusitis were detected in 30 sinuses (35.7%), 13 sinuses (15.5%), and 1 sinus (1.2%), respectively; in contrast, for the 310 sinuses with no AMO, mucosal thickening, a mucous retention cyst, and maxillary sinusitis were detected in 74 sinuses (23.9%), 35 sinuses (11.3%), and 7 sinuses (2.3%), respectively. Although the presence of the AMO was not found to be associated with a mucous retention cyst (*p* = 0.298) or maxillary sinusitis (*p* = 0.999), we detected a significant association between the presence of AMO and mucosal thickening (*p* = 0.029). In the binary logistic regression analysis to determine the factors influencing sinus pathologies, variables including age, sex, horizontal and vertical dimensions of the AMO, and antero-posterior and supero-inferior distance of the AMO from the NO were not found to be significant risk factors.

## 4. Discussion

In the present study, we three-dimensionally evaluated the location and dimension of the AMO and NO using CT images, as well as the spatial relationships between the AMO, NO, and several important adjacent anatomic structures. We also investigated the relationships between the presence of an AMO and maxillary sinus pathologies. The AMO was located posterior and inferior to the NO by an average of 5.4 mm and 0.7 mm, respectively, with a mean horizontal dimension of 2.8 mm and vertical dimension of 3.0 mm. In terms of the spatial relationship to adjacent anatomical structures, the AMO was only 1.3 mm below the orbital floor, but 30.2 mm above the maxillary sinus floor.

It is still not clear whether the AMO is a congenital anatomic variation or an acquired defect resulting from maxillary sinusitis [5]. However, several studies reported strong data supporting that the AMO are maxillary sinus perforations in the fontanelle caused by maxillary sinusitis [5,6,14]. Genc, et al. [5] showed the development of AMO following acute maxillary sinusitis in rabbit model. In addition, in the extensive endoscopic analysis by Mladina, et al. [6], an AMO was found more frequently in patients with chronic rhinosinusitis than in healthy subjects. Orhan Soylemez and Atalay [14] also suggested that the AMO may be an acquired defect caused from sinusitis or obstruction of the NO based on the significantly lower prevalence of the AMO in patients younger than 13 years. Similar to the tympanic membrane perforation in the otitis media, when the NO is obstructed due to severe maxillary sinusitis, perforation of the fontanelle, which has low resistance due to the lack of bony structures, may occur [6,14].

The prevalence of the AMO has been investigated through several clinical investigations using endoscopic analysis, cadaver analysis, and CT analysis, and has been reported to range in frequency from 0% to 47.2% [3,7,8,19]. May, et al. [15] examined the AMO by endoscopic investigation and reported a prevalence of 10%, while no AMO was observed in their cadaveric analysis. Serindere, et al. [23] also reported a similar prevalence of the AMO of 10.5% (42 of 400 patients) in CT examinations, with 19 patients (4.75%) exhibiting the AMO bilaterally. In contrast, several investigators reported higher prevalences of the AMO, as high as 45.5% in Yeung, et al. [24] and 47.2 % in Hung, et al. [8]. In the present study, the prevalence of the AMO was examined using CT data and was found to be 32.0% based on the number of patients and 21.3% based on the number of sinuses, that is, comparable to the results of previous studies. High between-study heterogeneity of the AMO prevalence may be caused by the analysis method and study design. While studies based on endoscopic and CT analysis reported similar prevalence of the AMO, cadaveric study reported a relatively low prevalence of the AMO [2,15]. This may be due to the small number of patients and difficulty in accurately detecting AMO, which are membranous defects with small diameter, after dissection in cadaveric analysis [14]. In addition, since most of the previous studies were conducted on patients who visited a single department such as oral and maxillofacial surgery or otolaryngology, the study population was different for each study, which may have affected the prevalence of the AMO. In CT analysis, the slice thickness of the CT scan and analysis method may also have been the cause of heterogeneity of the AMO prevalence. In terms of the influence of age or sex, no significant associations were found between age or sex and the presence of an AMO. Although Yeung, et al. [24] observed a significantly higher prevalence of the AMO in females, most previous studies reported results similar to our findings [7,19,25,26].

The AMO is usually located in the nasal fontanelle or hiatus semilunaris of the lateral nasal wall [3,10,15]. The fontanelle is the area between the uncinate process and inferior concha, which consists of two membranous layers, including the maxillary sinus mucosa laterally and nasal mucosal medially, without any bony structures [4]. Therefore, it may be the weakest region of the medial wall of the maxillary sinus, and it has been suggested that perforations from the sinus to the nasal cavity may result in the AMO [27]. Several investigators reported that the anterior nasal fontanelle was the most frequent location of the AMO, followed by the posterior nasal fontanelle and hiatus semilunaris [4,28]. However, May, et al. [15] reported that the AMO was found only in the posterior nasal fontanelle, posteroinferior to the NO. In a recent study by Hung, et al. [8], most AMO (81.1%) were located in the region of the nasal fontanelle or hiatus semilunaris, while 18.9% of the AMO were located outside the region of the nasal fontanelle and hiatus semilunaris. Unlike previous studies of the location of the AMO, in the present study, the distances between the AMO and adjacent structures, such as the maxillary sinus floor, orbital floor, and anterior maxillary sinus wall, which can be used as a reference for maxillary sinus-related surgery, were analyzed three-dimensionally.

The importance of assessing the anatomical location of the NO and AMO in relation to MSFA has been emphasized in several studies [18,29,30]. In MSFA, the maxillary sinus membrane is raised and bone substitute is grafted to the subantral space. For successful placement and maintenance of the dental implant, it is necessary to elevate the sinus membrane to the medial wall of the maxillary sinus so that a sufficient amount of bone can be grafted and the implant can be completely surrounded by regenerated bone [29]. However, the elevation of the membrane reaching the medial wall may affect the physiological function of the maxillary sinus, depending on the amount of elevation, location of the NO and AMO, and preoperative condition of the sinus membrane. In addition, postoperative edema and bleeding after MSFA can lead to thickening of the sinus mucosa, which may reach the NO or AMO [18,31].

The height from the maxillary sinus floor to the NO was reported to be 28.2 mm by Simsek Kaya, et al. [29] and 33.3 mm by Sakuma, et al. [18], which may not be a potential risk factor for postoperative pathologic changes of the sinus. In the present study, the height of the NO from the most inferior point of the maxillary sinus floor may also be considered to be sufficiently higher for prevention of decreased patency and obstruction of the NO; however, the height measured on the same coronal plane as the NO ranged from 19.4 mm to 45.6 mm, indicating it to be a potential risk factor in several cases when the NO is located relatively low. A low vertical dimension of maxillary sinus, excessively grafted bone substitute reaching to just below the NO, pre-existing mucosal thickening, and postoperative mucosal thickening can decrease the patency of or obstruct the NO or infundibulum, which can cause inflammatory or infectious processes by impairing ventilation and drainage of the sinus, leading to maxillary sinusitis [32,33]. In a study of edematous changes of the sinus membrane after MSFA, although mucosal thickening decreased to 1.7 mm on average at 9 months after surgery, it was 5.0 mm during one week after surgery, with a range of −4.0 mm to 29.6 mm [18]. In the same study, although the mean distance between the NO and the highest extension of the edematous changes was 10.3 mm, postoperative edema reached the NO in 8 of 72 cases. For the AMO, the exposure of the grafted material through the AMO to the nasal cavity has also been suggested as a possible risk factor for postoperative infection after MSFA [29]. Although the mean height of the AMO from the sinus floor was reported to be 21.9 mm by Simsek Kaya, et al. [29], 28.1% of AMO were less than 20 mm from the sinus floor. In the present study, the height measured on the same coronal plane as the AMO ranged from 14.5 mm to 42 mm, with a mean value of 27.3 mm; 12.2% (11 of 90) of sinuses were that less than 20 mm.

In the present study, the locations of the NO and AMO were analyzed based on the dentition to provide useful information for recognizing the location of the NO and AMO when performing sinus-related surgery through intraoral and transantral approaches. The NO was located above the upper second premolar in more than 50% of cases, followed by the upper first molar with a frequency of 29.5% and the upper first premolar with a frequency of 9.8%. Only one NO was located above the second molar. For the AMO, more than half of the AMO were located above the upper second molar, and the majority of the rest were located above the upper first molar, while there was no sinus where the AMO was located above or anterior to upper first premolar. Sakuma, et al. [18] also reported similar results, that the NO was located most frequently above the upper second premolars. In contrast, Simsek Kaya, et al. [29] observed that both the NO and AMO were located most frequently in the middle region, from the upper first molar to the upper second molar. The differences in dentition-related location may be explained by different reference planes in the 3D analysis using CT in each study.

The presence of an AMO may cause pathologic and morphological changes in the maxillary sinus mucosa through mucous re-circulation and defective mucociliary function due to a decreased concentration of nitric oxide [9,10,11,14,34]. Although the relationships between the presence of an AMO and various sinus pathologies have been assessed in several previous studies, they remain unclear. Several investigators reported significant relationships between the presence of an AMO and sinus pathologies [3,27,35]. In particular, Yenigun, et al. [3] found that the presence of an AMO was associated with a three-fold increase in the incidence of mucous retention cysts and a two-fold increase in the incidence of mucosal thickening and sinusitis. Ali, et al. [27] also reported consistent results, in which maxillary sinusitis was associated with the presence of an AMO. In that study, they regarded mucosal thickening as maxillary sinusitis and did not consider mucous retention cysts as a sinus pathology. Similarly, Orhan Soylemez and Atalay [14] found that mucosal thickening, maxillary sinusitis, and primary ostium obstruction were significantly more common in the sinuses with an AMO than in those without an AMO, while a mucous retention cyst was not associated with the presence of an AMO. In contrast, several studies reported no association between the presence of an AMO and sinus pathologies [8,36]. Hung, et al. [8] suggested that the length and area of the AMO, not the presence of an AMO, had a significant association with the morphological changes of the sinus mucosa. In the present study, although mucous retention cysts and maxillary sinusitis were not associated with the presence of an AMO, we found that mucosal thickening had a significant relationship with the presence of an AMO. However, age, sex, distance from the NO to the AMO, and horizontal and vertical dimensions of the AMO were not significant risk factors.

Previously, several research groups investigated the location and dimensions of the NO and AMO using CT images [18,19,20]. Most chose a single sectional image on each coronal, axial, or sagittal plane and performed measurements on single images. In contrast, in the present study, we defined each landmark three-dimensionally, obtained three-dimensional coordinates for each landmark, and calculated parameters related to the ostium itself and adjacent structures. However, this study has several limitations. First, the average age of the patients was relatively young and the sample was small. Considering that the AMO may develop as a result of maxillary sinusitis, the young age of the patients included in this study may affect the frequency of the AMO. Therefore, it is necessary to evaluate the frequency and location of the AMO and the relationship between the AMO and sinus pathologies in older patients. Second, the study population of this study was limited to patients who had chief complaints related to the oral and maxillofacial region. Although we excluded several pathologic conditions that may influence on the maxillary sinus, nasal or chronic rhinosinusitis symptoms could not be evaluated, which may have affected the prevalence of the AMO. Another limitation of this study is that a single investigator performed landmark determination. Therefore, to increase reliability of the results, measurements by at least two investigators and statistical tests for inter-observer reliability are necessary in future studies. In addition, although our results suggest a significant relationship between the presence of an AMO and mucosal thickening and the possibility that the AMO may affect sinus-related surgeries, such as maxillary sinus floor augmentation, it may be necessary to evaluate the actual clinical symptoms of mucosal thickening in the AMO and the effect of the AMO on postoperative outcomes to establish the clinical significance of these results.

## 5. Conclusions

The AMO exhibited a prevalence of 21.3% without any age and sex predilections and was located posteriorly and inferiorly from the NO by averages of 5.4 mm and 0.7 mm, respectively. Although the NO and AMO were usually located where they would not restrict sinus-related surgeries, such as MSFA, the NO and AMO were also found at lower positions that could affect postoperative physiologic function of the maxillary sinus and treatment outcomes. Therefore, for successful and safe treatment, it is necessary to perform careful assessment of the NO and AMO prior to surgery together with the evaluation of the status of the maxillary sinuses.

## Figures and Tables

**Table 1:** Landmarks and measurements used in this study.

	Definition
*Landmarks*	
AMO_sup	The most superior point of the AMO
AMO_inf	The most inferior point of the AMO
AMO_ant	The most anterior point of the AMO
AMO_post	The most posterior point of the AMO
NO_sup	The most superior point of the NO
NO_inf	The most inferior point of the NO
NO_ant	The most anterior point of the NO
NO_post	The most posterior point of the NO
MSF	The most inferior point of the maxillary sinus floor
MS_ant	The most anterior point of the maxillary sinus wall
OF	The most inferior point of the orbital floor
*Location of AMO/NO*	
AMO_AP location	Distance from the MS_ant to AMO_ant in the antero-posterior direction
AMO_SI location_inf	Distance from the MSF to AMO_inf in the supero-inferior direction
AMO_SI location_sup	Distance from the OF to AMO_sup in the supero-inferior direction
NO_AP location	Distance from the MS_ant to NO_ant in the antero-posterior direction
NO_SI location_inf	Distance from the MSF to NO_inf in the supero-inferior direction
NO_SI location_sup	Distance from the OF to NO_sup in the supero-inferior direction
*Dimension of AMO/NO*	
AMO_horizontal dimension	Distance from the AMO_ant to AMO_post in the antero-posterior direction
AMO_vertical dimension	Distance from the AMO_sup to AMO_inf in the supero-inferior direction
NO_horizontal dimension	Distance from the NO_ant to NO_post in the antero-posterior direction
NO_vertical dimension	Distance from the NO_sup to NO_inf in the supero-inferior direction

AMO, accessory maxillary ostium; NO, natural ostium.

**Table 2:** Characteristics of patients according to the absence or presence of an accessory maxillary ostium.

	Absence of an AMO	Presence of an AMO	*p* Value
Number of patients	134	63	
Sex			0.356 *
Male	69 (51.5)	28 (44.4)	
Female	65 (48.5)	35 (55.6)	
Age (year)	35.8 ± 17.6	37.7 ± 16.7	0.380 ^†^
Sinus pathologies			
Mucosal thickening			0.058 *
Absence	89 (66.4)	33 (52.4)	
Presence	45 (33.6)	30 (47.6)	
Mucus retention cyst			0.076 *
Absence	114 (85.1)	47 (74.6)	
Presence	20 (14.9)	16 (25.4)	
Maxillary Sinusitis			0.387 ^‡^
Absence	131 (97.8)	60 (95.2)	
Presence	3 (2.2)	3 (4.8)	□

AMO, accessory maxillary ostium. Data presented as number of patients (%) or mean ± standard deviation. * Chi-square test; ^†^ Mann–Whitney U test; ^‡^ Fisher’s exact test.

**Table 3:** Characteristics of the maxillary sinuses according to the absence or presence of an accessory maxillary ostium.

□	Absence of an AMO	Presence of an AMO	*p* Value
Number of sinuses	310	84	
Sex			0.187 *
Male	158 (51.0)	36 (42.9)	
Female	152 (49.0)	48 (57.1)	
Age (year)	35.9 ± 17.4	37.9 ± 17.0	0.272 ^†^
Sinus pathologies			
Mucosal thickening			0.029 *
Absence	236 (76.1)	54 (64.3)	
Presence	74 (23.9)	30 (35.7)	
Mucous retention cyst			0.298 *
Absence	275 (88.7)	71 (84.5)	
Presence	35 (11.3)	13 (15.5)	
Sinusitis			0.999 ^‡^
Absence	303 (97.7)	83 (98.8)	
Presence	7 (2.3)	1 (1.2)	

AMO, accessory maxillary ostium. Data presented as number of patients (%) or mean ± standard deviation. * Chi-square test; ^†^ Mann–Whitney U test; ^‡^ Fisher’s exact test.

**Table 4:** Location and dimension of the natural ostium and accessory maxillary ostium.

	Sex Differences			Overall
	Male	Female	*p* Value *	
*Location of the NO*				
NO_AP location	12.0 ± 2.4	10.9 ± 2.9	0.021	11.4 ± 2.7
NO_SI location_inf	35.2 ± 4.7	32.7 ± 4.2	0.009	33.8 ± 4.6
NO_SI location_sup	−3.5 ± 3.5	−3.0 ± 2.5	0.442	−3.2 ± 3.0
*Location of the AMO*				
AMO_AP location	21.9 ± 4.6	23.0 ± 3.5	0.517	22.5 ± 4.1
AMO_SI location_inf	31.5 ± 4.7	29.0 ± 5.0	0.025	30.1 ± 5.0
AMO_SI location_sup	1.0 ± 3.7	1.6 ± 3.8	0.587	1.3 ± 3.7
*Dimensions of the NO*				
NO_horizontal dimension	5.9 ± 2.9	5.9 ± 2.8	0.844	5.9 ± 2.8
NO_vertical dimension	4.7 ± 2.0	4.4 ± 1.9	0.347	4.6 ± 1.9
*Dimensions of the AMO*				
AMO_horizontal dimension	2.8 ± 1.3	2.8 ± 1.3	0.676	2.8 ± 1.3
AMO_vertical dimension	3.0 ± 1.2	2.9 ± 1.3	0.694	3.0 ± 1.2

NO, natural ostium; AMO, accessory maxillary ostium. Data presented as mean ± standard deviation. * Paired *t* test.

## Data Availability

The data presented in this article are available within this article.
